# Quality of death after elective surgery: a questionnaire survey for the bereaved family

**DOI:** 10.1186/s40981-023-00598-9

**Published:** 2023-02-06

**Authors:** Mariko Sato, Mitsuru Ida, Yusuke Naito, Masahiko Kawaguchi

**Affiliations:** 1 Department of Anesthesiology, Takatsuki General Hospital, Takatsuki, Japan; 2 Department of Anesthesiology, Nara Medical University, 840 Shijo-cho, Kashihara, Nara, 634-8522 Japan

**Keywords:** Perioperative death, Questionnaire, Quality of death, Functional disability

## Abstract

**Purpose:**

Postoperative death is the third leading cause of death in the world, but the quality of death after surgery has been poorly documented. This study aimed to evaluate the feasibility of a questionnaire survey for the bereaved family regarding the postoperative quality of death and the impact of preoperative functional disability on the quality of death.

**Methods:**

Patients aged ≥55 years who underwent scheduled surgery under general anesthesia in a tertiary-care hospital in Japan between April 2016 and December 2018 were enrolled. Patients’ functional disability was assessed using the 12-item World Health Organization Disability Assessment Schedule 2.0 (WHODAS 2.0) before surgery and scored based on the sum of the 12 items (0–48). Postoperative deaths were detected in medical records 3 months and 1 year after surgery. When death had occurred, a questionnaire on the quality of death using the short version of the Good Death Inventory (GDI) was sent to the bereaved family, which was scored as the sum of the 10 domains (10–70).

**Results:**

Of 4020 eligible patients, 148 patients (3.6 %) died within 1 year after surgery. A hundred and twenty-nine bereaved families were sent the questionnaire, and 83 of them (64.3%) submitted valid responses suggesting the high feasibility of this questionnaire survey. There were no differences between the GDI and WHODAS 2.0 scores (median 49 [interquartile range 41–55] vs. 49 [43–54], respectively, *p* = 0.90). In addition, multiple regression analysis of related factors using the short version of the GDI as a continuous variable showed that age and death in a facility other than that in which the surgery was performed were associated with lower GDI scores (*p* = 0.004 and *p* = 0.04, respectively).

**Conclusion:**

The completion rate was 64.3%. There was no association between the quality of death and preoperative functional disability; however, older age was associated with a higher quality of death, while death in a facility other than that in which the surgery was performed was associated with lower quality of death.

## Background

Advance in medical technology has decreased mortality after surgery, which is as low as 0.08 to 0.18% [1–5]. However, it is still an important issue and remains the third leading cause of death in the world [6]. Additionally, patient-reported outcome measures such as disability-free survival are attracting attention; however, this does not teach us about the status of patients who died postoperatively [7]. Although the importance of the quality of death has been described in the field of palliative medicine, and surveys on the quality of death have been conducted among the bereaved families of the deceased by using the short version of the Good Death Inventory (GDI), the quality of death after surgery has been poorly documented [8].

In recent years, biological age, represented by frailty or malnutrition, has been attracting attention for its association with postoperative morbidity and mortality in various types of surgeries [9–12], and this fact has been considered for shared preoperative decision-making, in which patients and their families decide whether to undergo surgery taking postoperative recovery in consideration [13–16]. In addition, a large number of instruments to measure frailty and nutritional status are available, and there is no consensus on how to assess these factors in preoperative practice. In contrast, the 12-item World Health Organization Disability Assessment Schedule 2.0 (WHODAS 2.0) is a generic assessment instrument for functional disability and its impact on postoperative outcomes (including chronic postoperative pain) has been validated across cultures and diseases [17–19]; however, studies investigating its association with postoperative quality of death are sparse.

This study aimed to conduct a questionnaire survey for bereaved families regarding the postoperative quality of death and to evaluate its association with preoperative functional disability.

## Methods

### Ethical approval

This is a secondary study of the prospective observational cohort study approved by the Institutional Review Board of Nara Medical University (Kashihara, Nara, Japan; Chairperson: Prof. M Yoshizumi; approval number 1141; 25 December 2015). Our initial study evaluated postoperative functional disability and its associated factors [9]. Informed consent was obtained preoperatively from all patients. This trial was registered with the University Hospital Medical Information Network Research Center (registration number UMIN000021671).

### Patients’ criteria

Patients aged ≧55 years who underwent scheduled surgery at Nara Medical University hospital between April 2016 and December 2018 were enrolled in the study. The exclusion criteria for our original study were as follows: patients requiring psychiatric treatment, patients who had previously been enrolled in this study (i.e., reoperation), or patients who required emergency surgery (defined as surgery performed within 24 h after being scheduled). This study included bereaved families of patients who died within 1 year after surgery.

### Data collection

Data on patient characteristics, including age, sex, the American Society of Anesthesiologists physical status (ASA-PS), and the 12-item WHODAS 2.0, were assessed. Intraoperative data, including operation time and types of surgery, were collected. Postoperative data, including place of death (our hospital, another hospital or institution, home, or unknown location), presence of palliative care, time elapsed between surgery and death, whether the patient died before or after hospital discharge, and the number of days until death occurred, were extracted from the medical records. The types of surgery were categorized according to the classification of the Japanese Society of Anesthesiologists. We provided the 12-item WHODAS 2.0 questionnaire and a stamped envelope to each enrolled patient 3 months and 1 year postoperatively. If there was no response, the research staff contacted patients or their families by telephone. Death after surgery was ascertained at 3 months and at 1 year by using medical records and responses from the bereaved family. When a death was verified, the questionnaire on the quality of death assessed by using the short version of the Good Death Inventory (GDI) was sent to the bereaved family [8]. This questionnaire survey was conducted by informing the bereaved family that there were no previous studies reporting on the quality of postoperative death and that, in order to improve the quality of death, we would investigate whether there were any associated factors that could affect the quality of death. Additionally, before sending a questionnaire, we confirmed that we could mail the questionnaire in consideration of the feelings of the bereaved family.

### Instruments

#### Short version of the GDI

The original version of the GDI features ten concepts that are usually considered important regardless of cultural background (the core 10 domains) and eight concepts whose importance depends on religious or cultural differences (the optional 8 domains) [8]. In this study, the short version of the GDI, in which only the core 10 domains are assessed (Table 1) with a score of 1 to 7 assigned to each question, was used to facilitate to answer. The final score is obtained by adding the individual scores from each question, and its value ranges from 10 to 70. A higher score is interpreted as evidence of a better quality of death.

**Table 1:** The short version of the good death inventory

A measure for evaluating good death from the bereaved family member’s perspective	
1 Being free from physical distress	
2 Being able to stay at one’s favorite place	
3 Having some pleasure in daily life	
4 Trusting physician	
5 Not being a burden to others	
6 Spending enough time with one’s family	
7 Being independent in daily activities	
8 Living in calm circumstances	
9 Being valued as a person	
10 Feeling that one’s life was completed	

The short version of the Good Death Inventory (GDI) is a questionnaire consisting of 10 domains and is used by the bereaved family to evaluate the condition of the patient before death. A score of 1 to 7 is assigned according to the answers to each question, and the sum of the scores for each item, 10–70, is used to evaluate the quality of death. A higher score is interpreted as a better quality of death

#### The 12-item WHODAS 2.0

The 12-item WHODAS 2.0 is a disability assessment tool with a recall period of 30 days. The validity of the Japanese version of the 12-item WHODAS 2.0 has been demonstrated in previous reports [18, 20, 21] (Table 2). The questionnaire consists of six domains of functioning (cognition—understanding and communicating; mobility—moving and getting around; self-care—hygiene, dressing, eating, and staying alone; getting along—interacting with other people; life activities—domestic responsibilities, leisure, work, and school; participation—joining in community activities) with 12 items. For each item, the patient has five choices; the score, depending on the choice, ranges from 1 (none) to 5 (extreme). Following the WHODAS 2.0 guidelines, the scoring system based on the item-response theory was adopted, and the score was modified by subtracting one point from the number of points answered. Therefore, the total score ranges between 0 and 48, with higher scores indicating higher functional disability. The score is expressed as a percentage (divided by 48 and multiplied by 100) in order to make it easier to understand [18]. The severity of the functional disability is based on the calculated score: none (0–4), mild (5–24), moderate (25–49), severe (50–95), and complete (96–100) [20]. We defined clinical disability as a 12-item WHODAS-2.0 score of equal or above 25.

**Table 2:** The 12-item world health organization disability assessment schedule 2.0

Assessment instrument that can measure health and disability	
1 Standing for long periods such as 30 min?	
2 Taking care of your household responsibilities?	
3 Learning a new task, for example, learning how to get to a new place?	
4 Joining in community activities (for example, festivities, religious, or other activities) in the same way as anyone else can?	
5 How much have you been emotionally affected by your health problems?	
6 Concentrating on doing something for 10 min?	
7 Walking a long distance such as a kilometer [or equivalent]?	
8 Washing your whole body?	
9 Getting dressed?	
10 Dealing with people you do not know?	
11 Maintaining a friendship?	
12 Your day-to-day work/school?	

The World Health Organization Disability Assessment Schedule 2.0 is a practical, generic assessment instrument that can measure health and disability at the population level or in clinical practice. The level of difficulty starts from “no difficulty” and increases in an ordered fashion to “mild,” “moderate,” “severe,” or “extreme” difficulty

### Outcomes

The primary outcome was the feasibility of the questionnaire survey. The secondary outcomes were the effect of preoperative functional disability on the short version of the GDI and its associated factors.

### Statistical approach

The feasibility of the questionnaire survey was evaluated as the response rate (the percentage of bereaved families who completed all of the items on the short version of the GDI). The Mann-Whitney *U* test was used to compare the short version of the GDI scores and preoperative functional disability. Multiple linear regression was performed to evaluate the factors associated to the answers to the short version of the GDI using all variables evaluated in the pre-, intra-, and postoperative periods. Continuous variables are expressed as median [95% confidence interval (CI)] or as value with percentage (Table 3). All statistical analyses were performed with EZR (Saitama Medical Center, Jichi Medical University, Saitama, Japan), and *p* < 0.05 was considered statistically significant. Since this study was a sub-analysis, sample size calculation was not performed.

**Table 3:** Patient demographic and medical information

Variables	Median [interquartile range] or number (%)
Preoperative variables	
Age (years)	74 [70–80]
Male sex	60 (72.3%)
ASA-PS	
1	4 (4.8%)
2	53 (63.9%)
3	26 (31.3%)
The weighting score of the 12-item WHODAS2.0	19 [6–47]
Functional disability assessed based on the score of the 12-item WHODAS2.0	38 (45.7%)
Intraoperative variables	
Operation time (min)	199 [76–327]
Types of surgery	
Neuro	5 (6%)
Thoracic	9 (10.8%)
Cardiac	4 (4.8%)
Esophageal	1 (1.2%)
Upper abdominal	21 (25.3%)
Lower abdominal	26 (31.3%)
Head, neck, pharyngeal, and laryngeal	8 (9.6%)
Chest wall, abdominal wall, and perineum	2 (2.4%)
Spine	2 (2.4%)
Hip joint and limbs	5 (6%)
Postoperative variables	
Place of death	
Our hospital	38 (46.3%)
Other hospitals	30 (36.6%)
Home	9 (11%)
Unknown	5 (6.1%)
Palliative care	22 (26.8%)
Died without discharge after hospitalization	5 (6%)
Days until death (days)	217 [139–303]

*ASA-PS* American Society of Anesthesiologists, ASA physical status classification; *WHODAS2.0* World Health Organization Disability Assessment Schedule 2.0

## Results

During the study period, 6060 patients were candidates for the study, and 4020 patients were eligible. One hundred and forty-eight patients (3.6%) died within 1 year of surgery, and the short version of the GDI questionnaire was sent to 129 bereaved families.

**Fig. 1:**
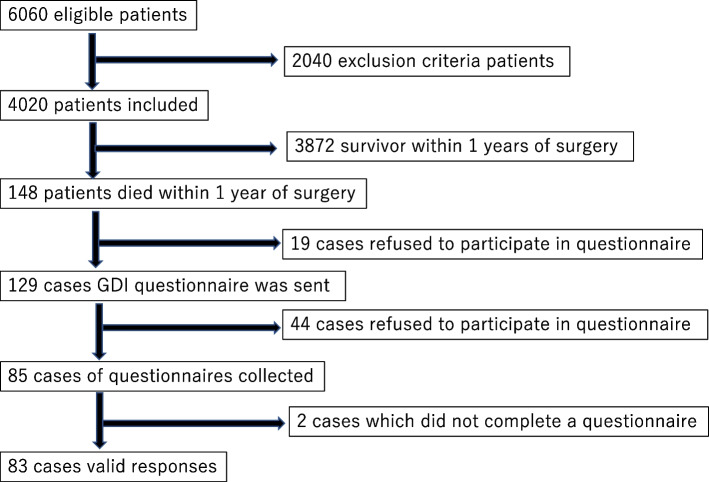
Patient flowchart for study inclusion and exclusion

**Fig. 2:**
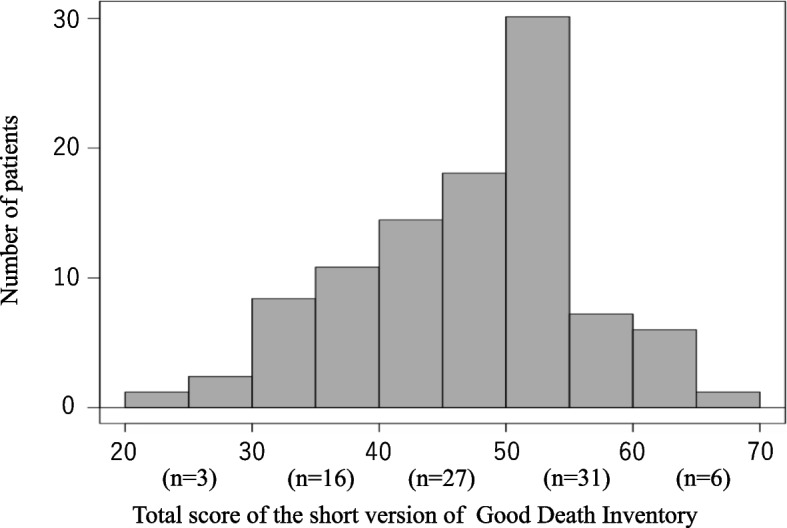
Histogram of the short version of the GDI score. The horizontal axis represents the short version of the GDI score, and the vertical axis represents the number of patients

Completed questionnaires were collected from 85 bereaved families (65.8%), and among them, the answers were considered valid in 83 cases (64.3%) (Fig. 1). The reasons for declining the questionnaire were “Death immediately after surgery,” “Offended by the questionnaire after death,” ”Too painful to reply,” “Too heavy crying when on the phone,” ”Dissatisfaction with treatment,” “Death found at home while living alone,” and address unknown. The most common site of surgery was the abdomen, and the most common cause of death was malignant neoplasm. The median patient age was 74 years [interquartile range (IQR) 70–80], sex was mostly male (72.3%), and the place of death was the hospital in approximately 83% of the cases. There were five patients who died without having been discharged from the hospital after surgery (Table 3). The results of the GDI score are shown in Fig. 2. The median for the GDI was 49 points, with the highest score being 68 and the lowest score 21. Out of the 83 patients for whom valid answers to the questionnaire were obtained, 38 patients (45.7%) had functional disability. There was no statistical significance between the short version of the GDI score and preoperative functional disability (median 49 [IQR 41–55] versus 49 [IQR 43–54], *p* = 0.90).

Multiple regression analysis, in which the score of the GDI was treated as a continuous variable, showed that age and death at institutions other than our hospital were significantly associated with the GDI score (*p*=0.004 and *p*=0.04, respectively, Table 4).

**Table 4:** Multiple regression analysis of the factors contributing to the Good Death Inventory (GDI) score

Association factors	Regression coefficient estimates	Standard deviation	*p* value
Age	0.40	0.14	0.004
Sex	−3.77	2.20	0.09
ASA-PS	0.21	2.29	0.92
The weighting score of the 12-item WHODAS2.0	1.95	2.19	0.37
Operation time	−0.008	0.006	0.17
Cause of death	−0.58	2.44	0.81
Died without discharge after hospitalization	4.15	5.01	0.41
Place of death			
Home	2.10	3.34	0.53
Other	−4.76	2.35	0.04
Unknown	1.88	4.30	0.66
Days until death	0.01	0.01	0.13
Palliative care	0.36	2.45	0.88

Multiple regression analysis of the short version of the GDI scores and related factors was also conducted to investigate factors influencing the short version of the GDI

*ASA-PS* American Society of Anesthesiologists, ASA physical status classification; *WHODAS2.0*, World Health Organization Disability Assessment Schedule 2.0

## Discussion

This study evaluated the feasibility of a questionnaire survey for bereaved families regarding postoperative death at a Japanese university hospital. Previous studies of bereaved families of patients with cancer or noncancerous who died in palliative care had response rates of 38%, 51%, and 56% [22–24]. This study response rate (64.3%) was higher than those from previous studies, showing that the survey was feasible. Moreover, this high response rate could have resulted from our specific efforts at follow-up; if there was no response, research staff contacted the bereaved family directly by telephone.

Our study did not reveal any impact of preoperative functional disability (assessed by the 12-item WHODAS 2.0) on quality of death (assessed by the GDI). Additionally, higher scores for the GDI were significantly associated with older age, whereas death in a facility other than the hospital where surgery had been performed was associated with lower scores (*p* = 0.04). Patients and their families can choose to undergo surgery depending on the preoperative functional disability of the patient and the risks associated with the procedure, some of which may result in postoperative death. Despite the fact that postoperative mortality is not low worldwide [6], no studies have assessed the quality of death after surgery. In this context, our study was the first to examine the association between preoperative functional disability and postoperative quality of death. Previous studies have reported that poor preoperative WHODAS 2.0 scores have a negative effect on the postoperative outcomes [9], although our study did not show any effect of preoperative functional disability (the 12-item WHODAS 2.0) on quality of death (the short version of the GDI). This may imply that the presence of functional disability before surgery does not prevent from undergoing surgery.

Older age was associated with a higher score of the GDI. The exact reason for this is unclear, but it has been reported in a previous study that young patients were significantly less likely to feel “one’s life was completed” and “being a burden to others” [25]; thus, younger persons might not achieve a good death.

Death in facilities other than the hospital where the patient underwent surgery was associated with a lower score of the GDI. The Ministry of Health, Labor and Welfare in Japan revealed that 69.2% of Japanese elders choose their home as their place to die. However, this fact has not been reflected in the real world: Only 13.6% died at their home, whereas 71.3% died at the hospital according to previous reports [26, 27]. Moreover, 94% of the Japanese population assumes that death at their home is not achievable because of the difficulty in receiving end-of-life care at home [28]. This could explain why most Japanese people are forced to undergo hospital admission due to severe physical symptoms, lack of caregivers, feelings of not wanting to be a burden for their family, and lack of home care clinics. In addition, previous studies on the place of death have uncovered that those who preferred a hospital were more likely to be female and older (≥75 years of age), make regular hospital visits, have experienced the death of a relative with cancer, have not given due thought to their own death, and be unfamiliar with home care nursing [29]. Taken together and considering the cultural background of the study population, these results indicate that death in facilities other than the hospital where surgery was performed may also help to explain the lower score observed in the short version of the GDI.

There were messages from 13 cases in the comment section of the GDI questionnaire. The messages were mainly expressions of gratitude, with some reflecting on their feelings towards the deceased, but also complaints about patient care. Cases with comments of gratitude tended to have a higher score of the GDI, while those with comments of dissatisfaction had lower scores. From the comments included in the GDI in the two patients with GDI scores of 27 and 37 (Table 5), it can be inferred that even if medical professionals are providing appropriate care and treatment, this may have not been appropriately communicated to the patients and their families. These comments can therefore be interpreted to mean that the patients and their families were made uncomfortable because of the lack of explanation by medical professionals, highlighting the importance of informed consent.

**Table 5:** Comments from bereaved family on the short version of the GDI questionnaire

Patient 1: GDI 68 points, 81 years, male, postoperative rectal cancer, died in another hospital 247 days after surgery due to the primary disease. He was transferred to another hospital because he had a history of amyotrophic lateral sclerosis (ALS), which worsened with the original disease. Comment: “Thank you very much for providing sufficient treatment for my late father at the end of his life. Thank you very much.”	
Patient 2: GDI 62 points, 69 years, male, cervical spinal cord injury in an accident, pulmonary embolism, died in hospital 18 days after surgery. He was independent in activities of daily living (ADL) before the operation, had a wish to prolong his life before his death, and applied for a donation. Comment: “This hospitalization helped me to reconcile with my son with whom I had a quarrel, to talk with my daughter about marriage and work, to talk about our 30 years of marriage on the day of the sudden change. I think my husband felt he was dying and was preparing to leave.”	
Patient 3: GDI 37 points, 82 years, male, abdominal aortic aneurysm, after hospital discharge and home nursing intervention, admitted to another hospital. He died 91 days after surgery due to interstitial pneumonia and was independent in ADL before surgery. Comment: “To keep the intravenous injection on, he had to wear gloves, be tied to a fence, and were constrictive clothing. I think it was very difficult for her both physically and mentally.”	
Patient 4: GDI 27 points, 61 years, male, septicemia from necrosis of the lower leg, tracheostomy, died in hospital 95 days after surgery. Comment: “It was hard watching the doctor give up through the tracheostomy. Some of the nurses talked to my husband and myself, while others just did their job in silence. I didn’t feel good about the way they called the feeding tube.” There were messages from 13 cases in the free comment section of the short version of the GDI questionnaire. The two cases with the highest and two cases with the lowest scores were selected from these.	

Although the study was performed rigorously, there are some limitations that should be considered when interpreting our results. First, our sample size was limited because this study was a secondary analysis. However, this study could be used as a stepping stone to initiate prospective studies at other healthcare centers. Second, the short version of the GDI in this study may have been overestimated because the reasons for refusal of the questionnaire were negative. Third, the population in this study was defined as those aged 55 years or older undergoing scheduled surgery. Thus, the scores may differ in cases of emergency surgery or when younger patients are considered. In the future, a multicenter study including a large patient population would be needed.

In conclusion, we conducted a questionnaire survey for bereaved families regarding postoperative quality of death in patients aged ≧55 years who underwent scheduled surgery under general anesthesia, resulting in a response rate of 64.3%. There was no significant association between the quality of death and preoperative functional disability; however, older age was associated with a higher quality of death, while death in a facility other than the hospital where surgery took place was associated with a lower quality of death. Further studies are required for the assessment of the quality of death in order to achieve a desirable death.

## Data Availability

They are available as a spreadsheet file upon reasonable request.

## Abbreviations

CI: Confidence interval
GDI: Good Death Inventory
IQR: Interquartile range
WHODAS: World Health Organization Disability Assessment Schedule
